# A Novel High-Affinity Potassium Transporter *IbHKT-like* Gene Enhances Low–Potassium Tolerance in Transgenic Roots of Sweet Potato (*Ipomoea batatas* (L.) Lam.)

**DOI:** 10.3390/plants11111389

**Published:** 2022-05-24

**Authors:** Wei Jiang, Rong Jin, Danfeng Wang, Yufeng Yang, Peng Zhao, Ming Liu, Aijun Zhang, Zhonghou Tang

**Affiliations:** 1 Xuzhou Sweetpotato Research Center, Xuzhou Institute of Agricultural Sciences, Xuzhou 221121, China; 20211013@jaas.ac.cn (W.J.); jinrong_2012@126.com (R.J.); 949270990@163.com (D.W.); zhaopeng0217@163.com (P.Z.); liuming0506@163.com (M.L.); zhangaijun608@163.com (A.Z.); 2 Cereal Research Institute, Henan Academy of Agricultural Sciences, Zhengzhou 450002, China; maybe483@foxmail.com

**Keywords:** sweet potato, *IbHKT-like gene*, potassium deficiency, transgenic roots

## Abstract

The high-affinity potassium transporters (HKT) mediate K^+^-Na^+^ homeostasis in plants. However, the function of enhancing low-potassium tolerance in sweet potato [*Ipomoea batatas* (L.) Lam.] remains unrevealed. In this study, a novel HKT transporter homolog *IbHKT-like* gene was cloned from sweet potato, which was significantly induced by potassium deficiency stress. *IbHKT-like* overexpressing transgenic roots were obtained from a sweet potato cultivar Xuzishu8 using an *Agrobacterium rhizogenes*-mediated root transgenic system in vivo. Compared with the CK, whose root cells did not overexpress the *IbHKT-like* gene, overexpression of the *IbHKT-like* gene protected cell ultrastructure from damage, and transgenic root meristem cells had intact mitochondria, endoplasmic reticulum, and Golgi dictyosomes. The steady-state K^+^ influx increased by 2.2 times in transgenic root meristem cells. Overexpression of the *IbHKT-like* gene also improved potassium content in the whole plant, which increased by 63.8% compared with the CK plants. These results could imply that the *IbHKT-like* gene, as a high-affinity potassium transporter gene, may play an important role in potassium deficiency stress responses.

## 1. Introduction

Potassium exists as a soluble ion (K^+^), which is not metabolized to an organic form in plants. K^+^ serves for many physiological processes, such as osmoregulation, turgor modulation, protein biosynthesis, control of membrane polarization, and transport of assimilation products [1,2,3]. Plant growth and quality are reduced when grown in soils containing limiting amounts of potassium nutrients [4,5]. The distribution of potassium resources is highly uneven globally. Moreover, there is a shortage of soil potassium and potassium fertilizer resources in China, which reduces the yield and quality of plant products. Previous studies showed that potassium deficiency affects the growth and development of the plant, particularly when it occurs in the initial phase of crop planting [6,7,8]. Therefore, it is important to increase the tolerance of plants to potassium deficiency. Genetic engineering is one method that can be used to improve abiotic stress tolerance in plants [9,10]. Transport characteristics for monovalent cations of high-affinity potassium transporters (HKT) are mainly attributed to their hydrophobic core structure with four repetitive transmembrane domain-pore domain-transmembrane domain (MPM) units. HKT transporters consist of two transmembrane regions and a conserved pore-forming domain (P-loop) and are arranged to form a central osmotic pathway [11,12]. According to the functional study and phylogenetic analysis of the heterologous expression system, the HKT family can be divided into two subfamilies in angiosperms. The ion selection characteristics of subfamily I and subfamily II are dependent on the amino acid at the first P_A_-loop conservative site of the HKT transporter. The amino acids at the four P-loop conservative sites of subfamily I, which are mainly present in dicotyledonous plants and have Na^+^ transport function, are Ser-Gly-Gly-Gly. The amino acids at the four P-loop conservative sites of subfamily II, which are mainly present in monocotyledonous plants, are Gly-Gly-Gly-Gly. Transporters of subfamily II are not only K^+^-Na^+^ cotransporters but also Na^+^ or K^+^ unidirectional transporter under different external environments [13]. The HKT members have been reported to adapt dicot plants to high-salt conditions by maintaining the K^+^-Na^+^ balance. Overexpression of *AtHKT1;1* gene in the root stele of transgenic *Arabidopsis* decreased the shoot Na^+^ content by 37–64% compared with the control. By contrast, *athkt1;1* mutant plants grew slowly as Na^+^ content increased, and the shoots were yellow. The *AtHKT1;1* gene moves more Na^+^ into the xylem parenchyma cells to maintain K^+^-Na^+^ homeostasis in *Arabidopsis thaliana* [14,15]. Overexpressing *SbHKT1* *gene* in cotton increased K^+^ uptake, K^+^-Na^+^ homeostasis, scavenging of reactive oxygen species, and salt tolerance [16]. Two HKT transporters in *Eucalyptus*, EcHKT1 and EcHKT2, mediated K^+^-Na^+^ cotransport in oocytes and compensated for K^+^ absorption defects in *E. coli* [17]. In monocotyledons, the HKT members can regulate K^+^ absorption under potassium deficiency. The *HKT1* gene expression level was rapidly and significantly increased in wheat roots when the external potassium concentration gradually decreased. *HKT1* gene expression was involved in the regulation of potassium ion absorption under low potassium stress [18,19]. A novel high-affinity potassium transporter *HvHKT7* gene has been found to respond to low potassium stress. However, no additional functional verification related to HvHKT7 has been published [20].

Sweet potato is an important food, feed, and industrial raw material [14,21], which requires high levels of potassium for optimum growth, and its yield and quality are restricted by potassium deficiency stress [22,23]. The development of low-potassium tolerant varieties is, therefore, an important objective of sweet potato breeders [24]. Genetic engineering is an effective method for enhancing the abiotic stress tolerance of sweet potatoes [25,26]. In a previous study, *IbHKT1* *gene*, which mediates K^+^ and Na^+^ uptake in *Saccharomyces cerevisiae*, was cloned and studied [27]. In our study, a novel high-affinity potassium transporter *IbHKT-like* gene was identified from the transcriptome data of sweet potato and cloned. Then, an *Agrobacterium rhizogenes*-mediated in vivo root transgenic system for sweet potato was used to overexpress the *IbHKT-like* gene. We found that overexpression of *the IbHKT-like* gene through this system enhanced the low-potassium tolerance in transgenic sweet potatoes.

## 2. Results

### 2.1. Identification of IbHKT-like Gene from Sweet Potato

**Figure 1:**
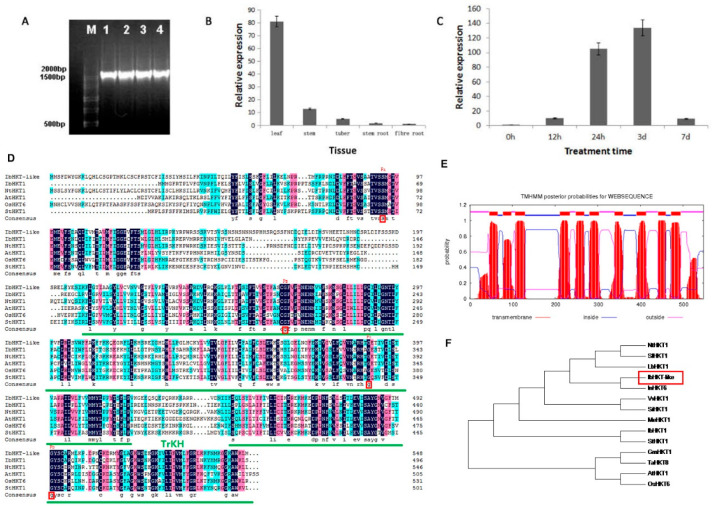
Identification of *IbHKT-like* gene and phylogenetic analysis. (**A**) Evaluation of amplified *IbHKT-like* gene sequence. (**B**) Expression pattern of *IbHKT-like* gene in different tissues of sweet potato. (**C**) Expression pattern of *IbHKT-like* gene in NZ 1 under low potassium stress. (**D**) Multiple sequence alignment and conserved domain analysis of IbHKT-like protein. (**E**) Prediction of the transmembrane domain of IbHKT-like protein, green lines are conservative domains, and red boxes are conservative sites. (**F**) Phylogenetic tree of HKT transporter with maximum-likelihood method. The ordinate is the relative expression level of the *IbHKT-like* gene. *IbARF* was used as the internal reference for data processing. Values indicated the mean and standard deviation of biological repetitions (*n* = 3).

An *IbHKT-like* gene was isolated from the transcriptome sequencing data of sweet potatoes. The ORF of this gene is 1647 bp in length and encodes a 548 aa polypeptide with 10 predicted transmembrane domains (Figure 1A,E). Multiple sequence alignment of this *IbHKT-like* gene and its orthologs from different species showed that the protein had two TrkH conservative domains, a subunit of the TRK system of the bacterial potassium transporter, between amino acids 200–412 and 435–535. The amino acids at the four P-loop conservative sites of IbHKT-like protein are Ser-Gly-Gly-Gly, which belong to subfamily I (Figure 1D). Phylogenetic analysis of the orthologs of HKT from different plant species indicated that this HKT gene from sweet potato is closely related to its orthologs in *Ipomoea nil* (InHKT6) but far away from *Arabidopsis* (AtHKT1) (Figure 1F). Thus, we named it *IbHKT-like gene*.

The expression level of *the IbHKT-like* gene was the highest in the leaf and the lowest in the fibrous root (Figure 1B). To assess whether the expression of the *IbHKT-like* gene in sweet potatoes is associated with potassium deficiency stress tolerance, a qRT-PCR was used to check the expression levels of the *IbHKT-like* gene in NZ 1 with low potassium tolerance. The expression of the *IbHKT-like* gene was significantly induced by potassium deficiency treatment in NZ 1. The low expression level was observed at the early stage (12 h) after treatment. With the extension of treatment time, the expression level of the *IbHKT-like* gene gradually increased and peaked at 3 d, which is 130 times that of 0 h (Figure 1C). The expression level of the *IbHKT-like* gene decreased at 7 d.

### 2.2. Generation of IbHKT-like Gene Overexpressing Roots in Sweet Potato

**Figure 2:**
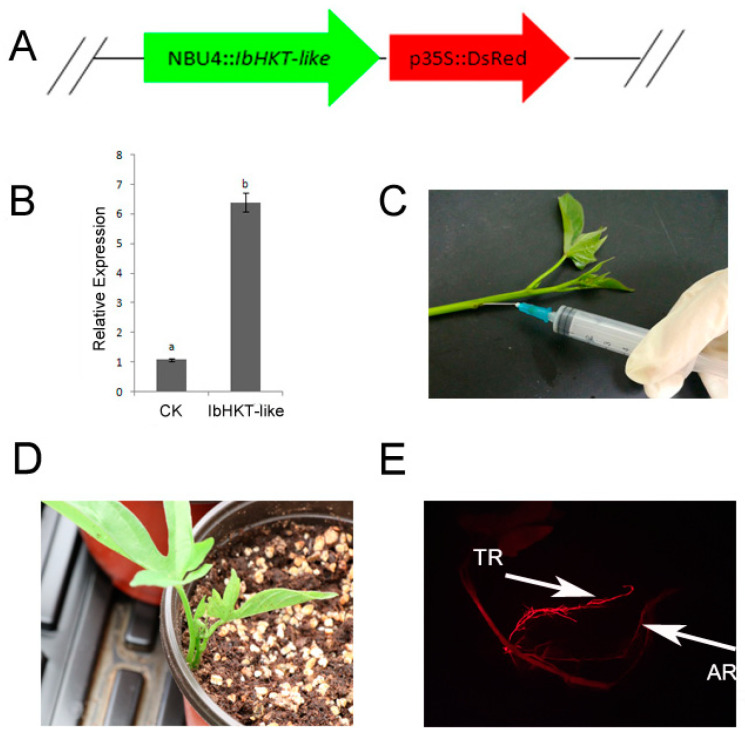
Generation of *IbHKT-like* gene overexpressing roots in sweet potato (**A**) NBU4::*IbHKT-like*-CaMV35S::DsRed expression cassettes. (**B**) Expression pattern of *IbHKT-like* gene in transgenic roots (TR) and non-transgenic roots, with relative expression of *IbHKT-like* gene on the ordinate. *IbARF* was used as the internal reference for data processing. Values indicated the mean and standard deviation of biological repetitions (*n* = 3): different letters indicated extremely significant *p* < 0.05. (**C**) Injecting sweet potato stem segments with *Agrobacterium rhizogenes* bacterial solution. (**D**) Seedlings grown in a pot after injection. (**E**) Identification of transgenic roots (with red fluorescent, TR).

To further validate the function of the *IbHKT-like* gene in sweet potatoes, we introduced the expression vector containing two expression cassettes (*IbHKT-like* gene + the DsRed marker) (Figure 2A) into *Agrobacterium rhizogenes* strain K599, which was then injected into the basal part of stems of uniform cuttings of sweet potato cultivars (Figure 2C). Several plants with *IbHKT-like* gene overexpressing roots in sweet potato were identified using the fluorescence detection system (Figure 2E). The qRT-PCR analysis showed that transgenic roots exhibited a significantly higher expression level (6.5 folds) of *IbHKT-like* gene compared with the CK (Figure 2B).

### 2.3. Protection of Cell Ultrastructure in IbHKT-like Gene Overexpressing Roots

**Figure 3:**
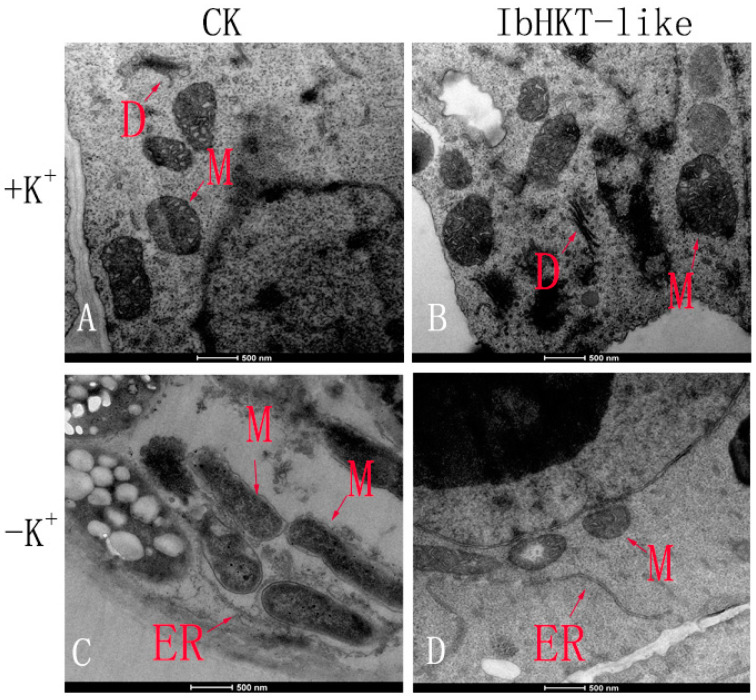
Effects of K^+^ deficiency on the ultrastructure of root tip cells (**A**) Ultrastructure on root tip cells of CK under control treatment. (**B**) Ultrastructure on root tip cells of overexpressing *IbHKT-like* under control treatment. (**C**) Ultrastructure on root tip cells of CK under potassium deficiency treatment. (**D**) Ultrastructure on root tip cells of overexpressing *IbHKT-like* gene under potassium deficiency treatment. +K^+^: 10 mmol·L^−1^ K^+^ treatment; −K^+^: 0 mmol·L^−1^ K^+^ treatment; CK: CK plants; *IbHKT-like*: transgenic root plants overexpressing *IbHKT-like* gene; ER: Endoplasmic reticulum; D: Golgi dictyosomes; M: Mitochondria. Bar = 500 nm (×11,000).

The ultrastructure of root tip cells under different K^+^ treatments was observed through TEM. A pronounced difference was shown in made images (Figure 3A–D). Under control (+K^+^) treatment, the root tip cells of both *IbHKT-like* transgenic and CK plants had a clear and round mitochondria, which are coated with a smooth outer membrane and an inner membrane [28], with obvious mitochondrial cristae, and intact and clear Golgi dictyosomes (Figure 3A,B). In *IbHKT-like* transgenic roots under potassium deficiency (−K^+^) treatment, the endoplasmic reticulum and mitochondria were intact and clear (Figure 3D). However, the root cells without overexpressing the *IbHKT-like* gene under-K^+^ treatment showed ruptured mitochondrial membrane and endoplasmic reticulum, blurred mitochondrial cristae, and the contents of mitochondria outflowed severely (Figure 3C).

### 2.4. Increase in K^+^ Absorption in IbHKT-like Gene Overexpressing Roots

**Figure 4:**
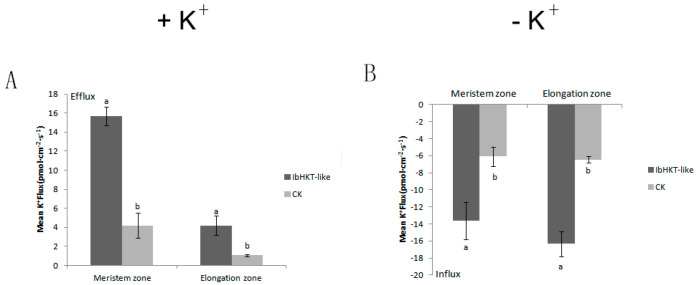
Steady-state K^+^ flow in CK and transgenic roots under different treatments. (**A**) Mean net K^+^ efflux rates in meristem and elongation zone of transgenic and CK roots. (**B**) Mean net K^+^ influx rates in meristem and elongation zone of transgenic and CK roots. The ordinate is the net K^+^ fluxes. Values indicated the mean and standard deviation of biological repetitions (*n* = 3): different letters indicated extremely significant *p* < 0.05. CK: CK plants; *IbHKT-like*: *IbHKT-like* overexpressing roots.

The effects of potassium deficiency on K^+^ flow in the root meristem and elongation zone of the *IbHKT-like* transgenic and CK plants were determined by noninvasive micro-measurement. The transgenic root tips and the control root tips treated with a K^+^ deficiency solution showed a trend of obvious K^+^ influx, and the amplitude of the transgenic root tip influx was significantly higher than that of CK. The average rates of meristem zone and elongation zone reached 13 pmol·cm^−2^·s^−1^ and 16 pmol·cm^−2^·s^−1^, 2.2 and 2.6 times that of the control, respectively (Figure 4A). Both *IbHKT-like* transgenic and CK root tips treated with a control solution showed an obvious K^+^ efflux trend, and the outflow amplitude of the transgenic root tip was significantly higher than that of the CK. The average rates of meristem zone and elongation zone reached 15 pmol·cm^−2^·s^−1^ and 4 pmol·cm^−2^·s^−1^, 3.8 and 4 times that of CK, respectively (Figure 4B).

### 2.5. Increased Potassium Content in Plants with IbHKT-like Gene Overexpressing Roots

**Figure 5:**
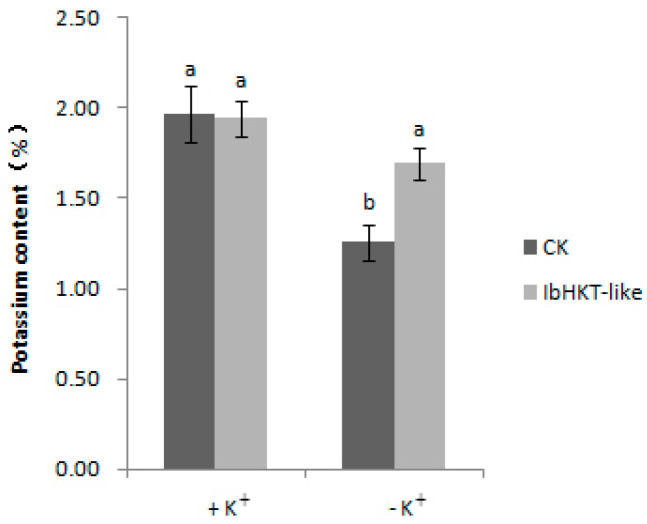
Total potassium content of CK plants and plants with *IbHKT-like* overexpressing roots under potassium deficiency treatments. The total potassium content of the plant shown as a percentage is on the ordinate. Values indicated the mean and standard deviation of biological repetitions (*n* = 8): the same letter indicated no significant difference; different letters indicated extremely significant *p* < 0.05. CK: CK plants; *IbHKT-like:* plants with *IbHKT-like* overexpressing roots.

To confirm if the *IbHKT-like* gene enhanced potassium deficiency tolerance, total potassium content was determined in *IbHKT-like* transgenic and CK plants treated with potassium deficiency and control solution. The dried plants were digested, and the potassium content of the whole plant was determined. The potassium content of plants in the control treatment was higher than that under the potassium deficiency treatment. There was no significant difference between the two under the +K^+^ condition. Under potassium deficiency treatment, the potassium content of the transgenic plants was at 1.69%, which was a significant increase (63.8%) compared with the CK plants (Figure 5).

## 3. Discussion

An *IbHKT-like* gene was isolated from the transcriptome sequencing data of sweet potatoes. The ORF of this gene is 1647 bp in length and encodes a 548 aa polypeptide (Figure 1A). Multiple sequence alignment of this *IbHKT-like* gene and its orthologs from different species showed that the protein had two TrkH conservative domains, a subunit of the TRK system of the bacterial potassium transporter, between amino acids 200–412 and 435–535, which means it has the function of transporting potassium ions. Moreover, the amino acids at the four P-loop conservative sites of IbHKT-like protein are Ser-Gly-Gly-Gly, which belong to subfamily I referring to previous studies (Figure 1D). *IbHKT-like* protein, with 10 transmembrane domains (Figure 1E), showed that it was significantly different from a previously reported *IbHKT1* protein sequence (containing 11 transmembrane fragments), with a similarity of 61%, and a long evolutionary distance (Figure 1F). IbHKT1 protein-mediated cotransport of Na^+^ and K^+^ in yeast. *IbHKT1* gene expression was highly induced by NaCl stress [29], while the *IbHKT-like* gene was highly induced by potassium deficiency stress. IbHKT-like protein has two TrkH domains (Figure 1D), which mediate K^+^ uptake in bacteria and probably evolved from simple K^+^ channels by multiple gene duplications or fusions, and is selective for permeation of K^+^ [30]. The hypothesis is advanced that *IbHKT-like* gene is involved in potassium nutrition and utilization. K^+^ plays an important role in field production and quality of sweet potato, which has a high demand for potassium.

Plants can perceive external K^+^ changes and generate chemical and physical signals, including ROS, in their cells. Mitochondria ultrastructure is closely related to ROS production [31,32]. The *IbHKT-like* overexpressing roots had stronger activity than that of the CK after 2–3 d of hydroponic culture under potassium deficiency conditions. Therefore, the ultrastructure of root meristem cells was observed. The ultrastructure of the CK treated with −K^+^ showed mitochondrial membrane rupture, blurred mitochondrial cristae, severe matrix outflow, and rupture of the endoplasmic reticulum. A variety of nutrient deficiencies led to the ultrastructure damage. Sulfur-deficient plants showed disintegration of the inner and outer membranes of mitochondria [33,34]. Potassium-deficient plants also showed distinct ultrastructural effects [35,36]. However, *IbHKT-like* overexpressing root meristem cells had intact mitochondria, endoplasmic reticulum, and Golgi apparatus. Therefore, it appears that the potassium transporter *IbHKT-like* gene protects cell ultrastructure from the damages of potassium deficiency stress. A complete cell ultrastructure is beneficial for potassium nutrition by the root system. K^+^ uptake by plant root cells is conducted by a large number of K^+^ channels and transporters [37]. K^+^ fluxes in root cells were detected, and there was an obvious K^+^ influx in treatments with −K^+^ solution. The amplitude of the transgenic root tip influx was significantly higher than that of CK. K^+^ uptake by roots was promoted by high-affinity potassium transporter under potassium deficiency, and more transporters were synthesized under long-term potassium deficiency stress (7 d) [38,39]. However, when treated with +K^+^ solution, K^+^ outflowed. The potassium deficiency solution used for measurement might be one reason. Also, *IbHKT-like* overexpressing root cells discharged more K^+^ than the CK, which may help maintain a balance between the potassium ion and other monovalent cations. Determination of potassium content showed that the transgenic plants had significantly increased potassium (63.8%) compared with the CK plants. This confirmed that overexpression of the *IbHKT-like* gene in sweet potato roots increased K^+^ uptake in K^+^ deficiency conditions. *MbtrkH* transgenic tobacco lines had significantly greater fresh weights, dry weights, and K^+^ contents compared with WT tobacco lines [40]. Potassium helps sorghum plants resist drought stress and improves the content of chlorophyll and carotenoids and the activities of SOD, CAT, APX, and nitrogen metabolic enzymes. The increase in potassium content in leaves promotes photosynthesis and reduces the loss of green [41,42]. In conclusion, overexpression of *IbHKT-like* gene through this system markedly protected cell ultrastructure, increased steady-state K^+^ influx, and improved potassium content in the plant.

## 4. Materials and Methods

### 4.1. Plant Materials and Growth Conditions

The sweet potato varieties used in this study were obtained from the Xuzhou sweet potato research center, Jiangsu, China. The sweet potato cultivar Xuzishu8 (XZ 8) was used for *IbHKT-like* overexpressing transgenic roots generation through infecting cut stems. The stems of XZ 8 collected from the greenhouse used to induce transgenic roots (TRs) through *Agrobacterium rhizogenes* were cultivated in pots (peat moss:loamy soil = 1:1) and placed inside a clean greenhouse at 25  °C and a 16:8 h (Light:Dark) photoperiod. Planting conditions of sweet potato cultivar Ningzishu1 (NZ 1) (sensitive to K^+^ deficiency stress) were the same as for XZ 8.

### 4.2. Gene Expression Analysis and Phylogenetic Analysis

Sweet potato cultivar NZ 1 (sensitive to K^+^ deficiency stress) seedlings were used to study the expression patterns of *IbHKT-like* gene in response to potassium deficiency stress for 12 h, 24 h, 3 d, and 7 d with hydroponic treatment, including improved Hoagland solution without potassium (potassium deficiency, −K^+^) and improved Hoagland solution (control, +K^+^). The solution was modified by Hoagland’s nutrient solution, containing 6.0 mmol·L^−1^ CaCl_2_, 6.0 mmol·L^−1^ NaNO_3_, 1.0 mmol·L^−1^ NH_4_H_2_PO_4_, 4.0 mmol·L^−1^ MgSO_4_, 0.2 mmol·L^−1^ FeSO_4_, 0.26 mmol·L^−1^ EDTA, 46 μmol·L^−1^ H_3_BO_3_, 10 μmol·L^−1^ MnSO_4_, 0.76 μmol·L^−1^ ZnSO_4_, 0.32 μmol·L^−1^ CuSO_4_, and 0.0162 μmol·L^−1^ (NH_4_)_6_Mo_7_O_24_. K^+^ treatment was set at two levels of 0 mmol·L^−1^ (−K^+^) and 10 mmol·L^−1^ (+K^+^) using KCl. The third leaves from the top, at all time points, were collected to extract total RNA using a TRIzol Reagent Kit. Total RNA was purified, and first-strand cDNA was synthesized using PrimeScript RT reagent Kit with gDNAEraser (Takara, Japan). The relative gene expression level was normalized to *IbARF* gene. The specific primers are listed in Table S1. The qRT-PCR primers were designed by Primer Premier 5.0. Quantitative real-time PCR (qRT-PCR) experiment with SYBR Green Realtime PCR Master Mix (ToYoba, Japan) was performed three times using a OneStep Real Time System (Applied Biosystems, Waltham, MA, USA). The experimental data were calculated by the 2^−^^ΔΔCT^ method [43]. The full-length amino acid sequences of IbHKT-like and its orthologs in other species were obtained from the UniProt and Phytozome databases. The amino acid sequences of the HKT protein were aligned by ClustalW with the default settings. The conserved sequences were underlined with a green line by DNAMAN (version 9.0, San Ramon, CA, USA). Transmembrane domains were predicted by TMHMM Server (version 2.0, Copenhagen, Danmark). A maximum-likelihood phylogenetic tree of HKT proteins was constructed using the MEGA program (version 7.0, Auckland, Zealand). The accession numbers of genes utilized in the present study are listed in Table S2.

### 4.3. Vector Construction and Agrobacterium Rhizogenes-Mediated Transformation

The coding region of *IbHKT-like* and DsRed was inserted into a pCAMBIA0390 expression vector. The overexpression construct *NBU4::IbHKT-like-p35S::DsRed* was used for *Agrobacterium rhizogenes*-mediated transformation. Uniform sweet potato (XZ 8) cuttings were collected for agroinfiltration using the following procedure. The construct was introduced into *Agrobacterium rhizogenes* strain K599 and cultured in 25 mL of LB liquid media consisting of 50 mg·L^−1^ kanamycin under shaking conditions (200 rpm) at 28 °C for 12–14 h. After the OD_600_ value reached 0.8, the cultures were centrifuged at 5000 rpm for 10 min at room temperature and then re-suspended in infiltration buffer (2 g·L^−1^ MES, 1 g·L^−1^ MgCl_2_, 200 μmol·L^−1^ AS; pH = 5.3) [44]. A 1.5 mL aliquot of the *Agrobacterium rhizogenes* suspension was then injected into the stem of sweet potato cuttings using a syringe with a needle. Afterward, the cuttings were transplanted into the soil and grown in a greenhouse using normal cultivation procedures. The transgenic positive plants, selected by a fluorescence detection system (LUYOR-3430-GR, San Francisco, CA, USA), were collected for analysis after 21–28 d of culture.

### 4.4. Observation of Ultrastructure of Root Tip

The transgenic positive and CK plants were selected for control and potassium deficiency treatments, respectively, with eight seedlings per treatment. Fine roots with 0.3 cm apices of adventitious roots (ARs) and TRs were collected from CK and *IbHKT-like* transgenic lines after potassium deficiency stress treatment for 96 h. Three seedlings were randomly selected from each replicate group. After treatment, the collected root meristematic zones were observed using an H-600 transmission electron microscope (TEM, Hitachi, Japan). The preparation of tissue specimens was referred to as the modified procedure [45].

### 4.5. Measurement of K^+^ Fluxes

Net K^+^ flux was measured using NMT (noninvasive microtest system, NMT-100-SIM-YG, Younger USA LLC, Amherst, MA, USA) as previously described [46,47]. Fine roots with 3 cm apices of ARs and TRs were collected from CK and *IbHKT-like* transgenic lines after potassium deficiency stress treatment for 96 h. K^+^ fluxes were monitored in potassium deficiency solution. Ion-selective microelectrodes with Nernstian slopes > 52 mV per decade for K^+^ were used. For K^+^ fluxes recording, the roots were rinsed with a potassium deficiency solution and immediately incubated in potassium deficiency solution to equilibrate for 10 min. Ion fluxes were determined along the root axis in two regions: the elongation zone (1–3 mm from the tip with a measurement interval of 500 µm) and the mature zone (10–15 mm from the tip with a measurement interval of 1 mm). Continuous recording was performed for 6 min at each measuring point in the two root zones. Steady-state ion fluxes were expressed as the mean of three measuring points in each root zone. The ion flux was calculated with JCal 1.0 (http://youngerusa.com/jcal, accessed on 15 March 2021).

### 4.6. Determination of Total Potassium Content

After potassium deficiency stress treatment for 96 h in a hydroponic way, CK and *IbHKT-like* gene transgenic lines were dried in an oven. The dried samples, including roots and shoots, were ground and digested with H_2_SO_4_-H_2_O_2_ [22], then potassium concentration was determined by flame photometry (FP640, Shanghai, China). Calculation of potassium content was made using the following formula: Total potassium (%) = ρ·V × 10^−4^/m.

ρ: the mass concentration of potassium found from the standard curve (mg·L^−1^); V: volume of measurement solution (mL); M: mass of dried sample (g); 10^−4^: conversion factor.

### 4.7. Statistical Analysis

Analysis of data presented as the mean ± SE was carried out with ANOVA using SPSS 20.0 Statistic Program (Armonk, New York, USA). Significance differences at *p* < 0.05 are indicated with different letters.

## 5. Conclusions

A novel HKT transporter homolog *IbHKT-like* gene, highly induced by potassium deficiency stress, was cloned. An *Agrobacterium rhizogenes*-mediated root transgenic system in vivo was used to generate overexpressing *IbHKT-like* roots in sweet potato cultivar Xuzishu8. Functional identification showed that overexpressing *IbHKT-like* gene protects cell ultrastructure, increases K^+^ influx, and improves the potassium content of plants. These results provide baseline information for advanced research on the molecular mechanism behind the *IbHKT-like* gene and the absorption and utilization of potassium.

## Data Availability

The data presented in this study are available in the graphs and tables provided in the manuscript and Supplementary Materials.

## Supplementary Materials

The following supporting information can be downloaded at: https://www.mdpi.com/article/10.3390/plants11111389/s1, Table S1: Primer information and function of *IbHKT-like* gene, Table S2: Accession numbers of HKT proteins from the different species.
