# Supplementary material for: A Novel High-Affinity Potassium Transporter IbHKT-like Gene Enhances Low–Potassium Tolerance in Transgenic Roots of Sweet Potato (Ipomoea batatas (L.) Lam.)
Source: Plants (Basel). 2022 May 24;11(11):1389. doi: 10.3390/plants11111389 (PMC9182616; doi:10.3390/plants11111389)
Supplement: Supplementary file 1 [file plants-11-01389-s001.zip › plants-1697310-supplementary.pdf]

Supplements

Table S1 Primer information and function of *IbHKT-like* gene

Gene	Upstream Primer (5′→3′)	Downstream Primer (5′→3′)	Function
IbHKT-like	ATGATGATGGGGTTCCGAAC	TTATGATAATTTCCAAGCTT	Gene clone
IbHKT-like	GTATTCCTGAGACTTTCCGTAT	AGCCCTGATGACTTCCAC	Real-time fluorescence quantitative PCR
IbARF	CTTTGCCAAGAAGGAGATGC	TCTTGTCCTGACCACCAACA	Internal reference

Table S2 Accession numbers of HKT proteins from the different species

Species	Gene	Accession number
Ipomoea nil	*InHKT6*	XP_019188746.1
Ipomoea batatas	*IbHKT1*	AMY98959.1
Nicotiana tabacum	*NtHKT1*	XP_016457809.1
Arabidopsis thaliana	*AtHKT1*	OAO98616.1
Manihot esculenta	*MeHKT1*	XP_021620110.1
Glycine max	*GmHKT1*	XP_006582258.1
Triticum aestivum	*TaHKT8*	ABG33945.1
Oryza sativa	*OsHKT6*	XP_015626193.1

Solanum tuberosum	*StHKT1*	XP_006359731.1
Vitis vinifera	*VvHKT1*	RVW85979.1
Sesamum indicum	*SiHKT1*	XP_011077901.1
Lycium barbarum	*LbHKT1*	AXY40149.1
Solanum lycopersicum	*SlHKT1*	NP_001295273.1
